# Physiological and biochemical variations of naturally ripened mango (*Mangifera Indica* L.) with synthetic calcium carbide and ethylene

**DOI:** 10.1038/s41598-024-52483-9

**Published:** 2024-01-24

**Authors:** Ashiq Hussain, Tusneem Kausar, Tahira Siddique, Khurram Kabir, Qurat Ul An, Farwa Rukhsar, Faiza Iftikhar Gorsi, Shazia Yaqub, Samina Kauser, Abdul Rehman, Ayesha Najam, Haseeb Haroon, Agbaje Rafiu, Sameh A. Korma, Amer Ali Mahdi

**Affiliations:** 1 Institute of Food Science and Nutrition, University of Sargodha, Sargodha, 40100 Pakistan; 2 Punjab Food Authority, Lahore, 54000 Punjab Pakistan; 3 Department of Food and Nutrition, Minhaj University Lahore, Lahore, 54000 Pakistan; 4 Food Processing and Value Addition Programme, Centre for Agricultural Development and Sustainable Environment, Federal University of Agriculture, Abeokuta, Nigeria; 5 Department of Food Science, Faculty of Agriculture, Zagazig University, Zagazig, 44519 Egypt; 6 School of Food Science and Engineering, South China University of Technology, Guangzhou, 510641 China; 7 Department of Food Science and Nutrition, Faculty of Agriculture, Food and Environment, Sana’a University, Sana’a, Yemen

**Keywords:** Biochemistry, Physiology

## Abstract

To meet the increasing consumer demands for fruits, the implementation of artificial ripening techniques using synthetic chemicals has become increasingly commonplace among less ethical fruit production companies in today’s global market. The objective of present work was to establish a difference in the physiological and biochemical and profiles of naturally ripened mangoes vs. those ripened by application of synthetic calcium carbide and ethylene. The application of calcium carbide at 10 g/kg mangoes resulted early ripening in 2 days, with a 3-day shelf life, as compared with 5 and 6 days, for mangoes ripened by ethylene and naturally, respectively. Higher levels of calcium carbide reduced moisture, fiber, protein and carbohydrates content and increased the ash content of mangoes, as compared to higher levels of ethylene, whereas in naturally ripened mangoes the content percentages were 80.21, 3.57, 3.05 6.27 and 4.74, respectively. Artificial ripening resulted in significant loss of ascorbic, citric and malic acid, as values were recorded 35.94, 2.12 and 0.63 mg/g, respectively, in mangoes ripened with 10 g/kg of calcium carbide. However, in naturally ripened mangoes the amounts of these acids were recorded significantly (*p* < *0.05*) high as 52.29, 3.76 and 1.37 mg/g, respectively. There was an increase in total soluble solids (TSS) and reducing sugars, and a decrease in titratable acidity in calcium carbide (10 g/kg) treated mangoes. Elemental analyses revealed high levels of minerals in naturally ripened mangoes, with significant values of iron (0.45 mg/100 g), zinc (0.24 mg/100 g) and copper (0.17 mg/100 g). The organoleptic quality of the fruit decreased significantly (*p* < *0.05*) as a result of the use of calcium carbide. Although use of artificial ripening techniques provides speedy ripening of mangoes, there are obvious limitations. Consequently, natural ripening should be promoted in order to have safer and more nutritious mangoes.

## Introduction

Safe and nutritious food in recommended daily allowances for one’s age range is a basic requirement for overall good health. Populations without access to safe and healthy foods suffer from a variety of associated health complications. It is well-known that fresh fruits and vegetables are, overall, the healthiest food for people of all ages due to the essential nutrients that they provide^1^. Universal demand for mature ripened fruits has increased due to the growing global population. However, the lack of adequate agricultural facilities and postharvest techniques has increasingly resulted in the use of cheaper, unhealthy, banned and toxic chemicals for ripening of fruits, in under-developed and developing countries. This state of affairs has thus alarmed many producers and market sellers with regard to safely meeting the needs of today’s more health-conscious consumers^2^.

Emerging populations need fresh fruits and vegetables in bulk, but due to the perishable nature of fruits and vegetables, their post-harvest losses are out of control. As a result, there is huge pressure on industries to supply fresh fruits and vegetables to the consumers. This pressure on industry could be reduced by encouraging the use of food grade fruit ripening and preserving agents, and health and environment friendly technologies to increase the shelf life of fruits and vegetables^3^. Fruits attain their pleasing taste and attractive color by the process of ripening. Nowadays, a considerable number of fruits found in the market are ripened by artificial methods. Dangerous chemicals are being used in this artificial ripening process, which are detrimental to health^4^. Changes in physicochemical, nutritional and organoleptic parameters of mangoes, throughout application of different post-harvest handling and artificial ripening techniques, will allow the consumers, producers and market sellers, to select the best fruits that can add value to their healthy lifestyle^5^ Use of calcium carbide in ripening of mangoes results serious health problems like ulcers, neurological disorders, hypoxia and memory loss. Identification of calcium carbide as ripening agent used, and quantification of this carcinogenic compound is very necessary for prevention of health issues. Therefore, different modern technologies have been developed recently, to encounter such lethal activities^6^. Instead of utilization of calcium carbide, different novel and friendly techniques have been discovered, which provides good quality fruits with uniform physical parameters^7^.

In today’s modern and fast lifestyle, rapid and fast ripening of fruits through synthetic agents, has become a trend, which should be restricted to preserve the quality and nutritional status of the fruits, during post-harvest period^8^. Climacteric fruit’s ripening has been found associated with the changes in fruit’s gas composition, due to speedy respiration leading to ethylene production. Different literatures have conflicts in the results, about gas production in attached and detached fruits. Pre-harvest and post-harvest factors produce significant differences in ripening duration, physical, chemical and nutritional contents, and shelf life of the mangoes^9^. Mango has been listed among the most cultivated tropical fruits, processed, shipped, preserved and marketed in the whole world. Therefore, investigations are very necessary, about the different ripening methods, and their effects on organoleptic and quality parameters of ripened mangoes^10^.

Mango (*Mangifera indica* L.), known as the king of fruits, has an attractive taste and fragrance, and high nutritional value. Pulp is the most-consumed part, while the peel and kernel are usually discarded. Mangoes contain a blend of sugars (16–18% *w*/*v*) and acids, and high amounts of antioxidants (ascorbic acid) and polyphenols (carotene, as vitamin A). The principal carbohydrates that are used are different, in green unripe and matured ripe mango. Sufficient quantities of macro and micro elements are also present in mangoes, making it a nutritious food for all age of people^11^. Mangoes are grown in tropical and sub-tropical regions of Asia, Africa and America. Worldwide, Pakistan is ranked fifth largest mango producing country, with over 300 varieties, including famous cultivars like Chaunsa, Sindhri, Anwar-Retol, Langra and Dusheri. In mangoes, natural ripening has been linked with some limitations, such as poor keeping quality, reduced shelf life and improper post-harvest processing^12^. Safe, controlled, and regulated ripening of mangoes, through authorized techniques/protocols, will not only add value to this fruit, but will also improve nutritional quality.

The rapid ripening process of mangoes, with the use of calcium carbide, has been found linked with exogeneous acetylene gas production, as a result of moisture gained by calcium carbide from fruits surface, and triggering the ripening process. Commonly used as a cheap artificial ripener also named as “masala” has gained economic stunt, although it has been banned in several countries, but still its use is common in running and shady markets^13^. Experiments were conducted by Abubakar^14^, to analyze the effect of the different concentrations of calcium carbide as ripening agent, on trace elements present in mangoes, and traces of arsenic and phosphine were reported in such artificially ripened mangoes, because industrial grade calcium carbide contains arsenic and phosphorus. Some of the health risks found associated with use of such calcium carbide ripened mangoes have been reported as, inability of body to resist infection, imbalance of hormones, and dysfunction of male reproductive system^15^.

Although some earlier studies have confirmed the deleterious effects of calcium carbide ripened mangoes, due to the presence of toxic chemicals, but these were not sufficient to provide detailed chemical, organoleptic and nutritional attributes of mangoes, ripened by the applications of different techniques, in a comparative mode. Keeping in view the research gaps, the present research experiments were designed to assess the harmful effects of calcium carbide as ripening agent and healthful impacts, of natural and ethylene-based ripening of mangoes. Ripening time, shelf-life analysis, physicochemical and organoleptic profiles of differentially ripened mangoes were compared to assess the best ripening technique, that could provide the required quality of fruits with health promoting potential.

## Materials and methods

Use of plants in the present study complies with international, national and/or institutional guidelines.

### Fruit material and post-harvest treatments

Mango (*Mangifera indica* L.) fruits (variety Chaunsa), at mature green stage were harvested from 10 to 12 years old healthy tree from Haji Laal Din mangoes farm, located in district Sargodha, Province Punjab, Pakistan. During harvesting of mangoes for further research work, key factors considered were uniformity of size, weight, color, shape and firmness. Fruits were selected for absence of disease, injury, bruises and physical damage. Harvested mature mangoes were carefully handled during field heat removal for some hours and were shifted into cartons for immediate transportation to laboratory, within one hour, via road transportation, through controlled temperature vehicle. Fruits chosen for experiments in the laboratory, were subsequently subjected to transient surface disinfection using NaClO solution (0.5%, v/v), rinsed with tap water and dried.

### Experimental design and treatments

Afterwards, mango fruit were randomly divided into 7 treatment groups replicated thrice and 12 mangoes were placed in each corrugated fiberboard box of uniform size and dimensions. In first experimental design three groups of calcium carbide treated mangoes were developed, as in 1st group calcium carbide was applied at concentration of 1 g/kg mangoes, in second group 5 g/kg mangoes and in 3rd group 10 g/kg mangoes. In this experimental design, calcium carbide bags were placed at the bottom of each container, in which mangoes were heaped for artificial ripening. Ventilated corrugated cartons made from food grade tertiary packaging material were used for mangoes ripening and guidelines described by Andrew et al.^16^ were followed, with some modifications.

**Figure 1:**
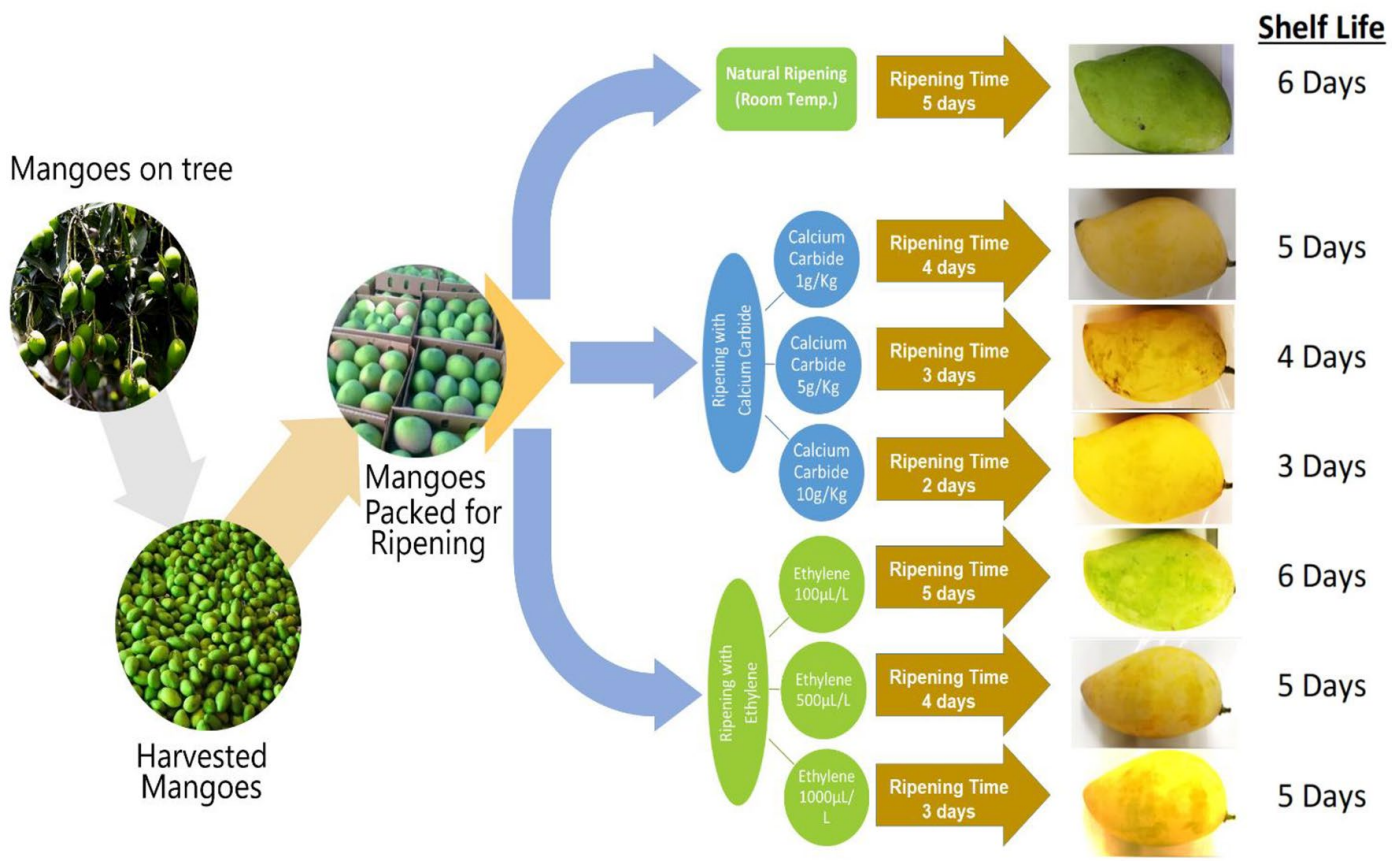
An overview of the experimental design.

In second experimental design 3 groups were developed to whom commercial certified ethylene was applied as ripening agent, by following the guidelines explained by Montalvo et al.^17^. In 1st group of this experimental design, concentration of exogenous ethylene was 100 µL/kg mangoes, in second group 500 µL/kg mangoes and in 3rd group 1000 µL/kg mangoes. While in 3rd experimental design control mangoes were allowed to ripen in controlled conditions without use of any commercial ripening agent. Room temperature of 25 ± 2 °C and relative humidity 85–90% was maintained during the whole completion of ripening under all treatments. After the completion of ripening process of each group, the mangoes were selected for further physicochemical, biochemical and organoleptic analyses. An overview of the research work, in the form of graphical image has been presented in Fig. 1.

### Chemicals and reagents used

Industrial grade calcium carbide was purchased from local welding shop located in district Sargodha, Pakistan, for use to ripen mangoes artificially and quantities were weighed in analytical lab weighing balance (PW 184 Adam, China), and small paper bags were packed according to treatments plan. Certified ethylene was supplied by Praxair (Praxair, Mexico S.A. de C.V.). Ethylene concentration in the chamber was checked by GC. Other chemicals, reagents and solvents required for this research work were of analytical grade purchased from DTS fine chemical limited, Islamabad, Pakistan.

### Ripening time and shelf-life analyses of mangoes

Ripening time of different treatment mangoes was carefully observed as an indicator of color development during ripening process, after regular interval of times. Farmers and market sellers use color development as key indicator of mango ripening, and yellow to orange color is clear indication of fully ripened mangoes. Further penetrometer was used to detect the soft texture developed in fully ripened mangoes as guided by Andrew et al.^16^.

Shelf-life analysis of naturally ripened mangoes and mangoes ripened artificially by use of calcium carbide and ethylene, was carried out by observing the mangoes fitness for use, at regular intervals of times after the completion of ripening time. From the first day of storage until the mangoes reached the stage when the fruits become unfit for consumption, has been calculated as shelf-life of mangoes.

### Determination of proximate composition of mangoes

The Association of Official Analytical Chemist’s^18^ approach was used for the proximate analyses of mangoes. The following were the official AOAC techniques, which were adopted for analyzing the different parameters: crude ash 942.05 (4.1.10), moisture 934.01 (4.1.03), crude protein 955.04 (2.4.03), crude fiber 962.09 (4.6.01), and crude fat 920.39 (4.5.01). Whereas, for carbohydrates all these values were summed and subtracted from 100. Each analysis was conducted in triplicate, after which mean values of the results were taken for further analysis.

### Total soluble solids, titratable acidity, reducing sugars, ascorbic acid, citric acid and malic acid contents of mangoes, naturally ripened, ripened with calcium carbide and artificial ethylene

#### Total soluble solids

Using dissimilar methods, samples of pulp tissue weighing about 15–20 g were taken from each treatment mango fruit individually, and afterwards liquified, filtered and centrifuged (10,000 rpm, 10 min) with extractor of commercial juice. The TSS was then determined from unsubstantial juice using the equipment named Atago PR-1 refractometer and expressed as Brix of the juice, following the method ratified by Schmilovitch et al.^19^.

#### Titratable acidity

The mango pulp specimens weighing about 15–20 g were obtained by using various techniques from each mango individually and were squashed, filtered and centrifuged (10 min at 10,000 rpm). The sheer juice was used for determining titratable acidity. The sheer juice was titrated with 0.1 N NaO7776H to determine titratable acidity and the data obtained was expressed as percentage acidity in the juice. The entire experiment was conducted following the methodology, as used by Schmilovitch et al.^19^.

#### Reducing sugars

In the methodologies adopted by Lima et al.^20^, some modifications were made for evaluating the reducing sugars in different mango samples. Stating briefly, using the tissue homogenizer at 25–28 °C, 5 g of pulp from each category was homogenized in 5 mL, 0.5 N of NaOH and 50 mL water and then was neutralized with 0.5 N of acetic acid, the final volume was made 100 mL after standardization, mixing and filtration. Afterwards, 20 mL of 85% of ethanol and 5 mL of extract were homogenized. The mixture slurry was centrifugated at 4 °C (25,000*g* for 20 min) to separate the supernatant, which was then removed. The mixture of extract was evaporated using double boiler at 65 °C until the extract volume remained 5 mL, and these residues were washed with distilled water. The contents of reducing sugars were analyzed by using the resulted extract. The d-glucose standard curve was used for evaluating the amounts of reducing sugars.

#### Ascorbic acid

Mango chunks obtained from different treatment samples ripened by application of different techniques were titrated against indophenol dye (2,6 dichloro indophenol, sodium salt) for the evaluation of contents of ascorbic acid, as illustrated by Sogi et al.^21^. Briefely describing, 10 g of smashed mangoes and 25 mL extraction solution (15 g meta-phosphoric acid: 40 mL acetic acid: 3.7 mL conc. sulfuric acid: 450 mL water) were homogenized and placed in water bath equipped with shaker for at least 1 h duration at 22 ± 1 °C. The remains were extracted twice along with the 10 mL extraction solution and then centrifuged vertically (10,000*g* for 5 min), un till unless, the pink color last for at least 15 s, the supernatants from each treatment were titrated against the dye solution (containing 50 mg dye, 42 mg NaHCO_3_, and 200 mL water). By titrating against dye, with fresh ascorbic acid solution as standard, the dye factor was calculated after each analysis.

#### Citric acid and malic acid

##### Samples preparation

For determination of citric acid and malic acid, 5 g of pulp from each treatment mango was taken and homogenized in 25 mL double distilled water. These homogenized mixtures were centrifugated at 3000 rpm for 30 min. After centrifugation, sediments were washed with 25 mL distilled water and supernatant after centrifugation was collected and pooled. Sep-Pak C18 filters were used to filter the supernatants and 10 mL were passed from a cation exchange column (50–100 mesh), and then hydrated with distilled water to make 25 mL total volume, which was filtered through a 0.45 µm membrane then poured in vials and stored at − 20 °C for further HPLC analysis as elaborated by Tovar et al.^22^, with slight modification.

##### HPLC analysis

For HPLC analysis guidelines of Tovar et al.^22^ were adopted with some changes. Briefly stating, volumes of samples injected were 100 µL and two reverse phase Bondclone-10 C18 columns with 0.5 µm pore size were connected in series at room temperature, and were used to determine organic acids in different treatment mango samples. Mobile phase used in this analysis was 0.05 M KH_2_PO_4_ and pH was set 2.2 with H_3_PO_4_, whereas 0.4 mL/min flow rate was adjusted. For detection photodiode array detector was fitted at 214 nm in liquid chromatography system. External standards were used for quantitative assessment and three replicates were performed for each sample treatment.

### Organoleptic parameters

Organoleptic parameters of naturally ripened mangoes and mangoes ripened with use of commercial calcium carbide and ethylene were analyzed by using 9-point hedonic scale as described by Larmond^23^. Briefly describing critical scores on the proposed proforma were noted from a panel of 80 judges from both the genders, with an average age of 35 to 40 years on randomly cut mangoes into 6 to 7 cut pieces. Each person present in separate booth was provided separate sheets and distilled water for mouth rinsing after each trial.

### Mineral analysis

Mineral analysis of different treatment mangoes was carried out through atomic absorption spectrophotometer (Varian AA240, Australia), equipped with hollow cathode lamps, by following the procedure adopted by Hernandez-Sanchez et al.^24^, with some modifications. Fully ripened mango fruits were peeled off and then samples were cut into small pieces with the help of uncontaminated steel knife. Dry ashing method was adopted and all samples were handled with nitrile gloves. Each 30 g sample of mango pulp was quartered and homogenized and then shifted to previously weighed porcelain capsules. The capsules containing mango pulp samples were dried in hot air oven at 65 °C for 24 h first, and then these samples containing crucibles were introduced in muffle furnace at 450 °C to free the sample from any organic matter and dried white ash obtained was dissolved in 5% nitric acid to make volume 50 mL and then this digested mixture was transferred in to 100 mL polyethylene bottles for further analysis.

### Statistical analysis

All results in this investigation are presented as means ± SD, and every trial and analysis was done in triplicate. Influence of different ripening protocols on different parameters of mangoes was noted, and the data from the experiments were statistically evaluated using Tukey’s post hoc test and ANOVA with a 95% confidence level, using JMP software (Version 10.0, SAS). P-values were employed to evaluate the importance of the parameters under study's influence.

## Results

### Ripening time and shelf-life analysis of mangoes; naturally ripened, ripened with calcium carbide and artificial ethylene

Comparison of different ripening techniques used for mangoes has been presented in terms of ripening time and self-life of mangoes in Table 1, and from data significantly different results could be seen, as natural ripening although taken 5 days’ time to ripen but shelf life of mangoes was prolonged to 6 days. Higher concentration of calcium carbide (10 g/kg) induced fast ripening of mangoes but shelf life was reduced to 3 days only. On the other hand, ethylene application at 1500 µL/L forced mangoes to ripen in 3 days and shelf life was also increased to 5 days. From the observations of shelf-life study, it can be concluded that natural ripening helps to prolong the shelf life of mangoes during post-harvest storage and transportation, while ripening with calcium carbide and ethylene decreases the shelf life of the mangoes, which possibly was due to the faster respiration and metabolic activities in the tissues.

**Table 1:** Ripening time and shelf-life analysis of mangoes, naturally ripened, ripened with calcium carbide and artificial ethylene.

Ripening time and shelf-life analysis of mangoes ripened through different techniques			
(a)	Concentration (g/kg)	Ripening time (days)	Shelf-life (days)
Mature mangoes ripened with calcium carbide	1	4	5
	5	3	4
	10	2	3

(b)	Concentration (µL/kg)	Ripening time (days)	Shelf life (days)
Mature mangoes ripened with artificial ethylene	500	5	6
	1000	4	5
	1500	3	5

(c)	Ripening time (days)	Shelf life (days)	
Mature mangoes ripened naturally	5	6	

### Proximate composition of mangoes, naturally ripened, ripened with calcium carbide and artificial ethylene

Proximate composition of mangoes naturally ripened, ripened with different levels of calcium carbide and ethylene, has been presented in Table 2. Ripening with calcium carbide, at increased concentrations (10 g/kg) caused significant (*p* < *0.05*) reduction in moisture, fiber and protein contents, and significant (*p* < *0.05*) increment in ash, fat and carbohydrates of the mangoes more significantly as compared to natural ripening and ripening with lower levels of ethylene. Moisture content in naturally ripened mangoes was 80.21 ± 0.09%, which was reduced to 77.63 ± 0.06% in ethylene ripened, and further 75.85 ± 0.06% in carbide ripened mangoes. Contents of ash were found higher (5.46 ± 0.01%) in carbide ripened mangoes, as compared to ethylene ripened mangoes (5.15 ± 0.02%) and naturally ripened mangoes (4.74 ± 0.03%). On the other hand, lower carbohydrates were calculated in naturally ripened mangoes with value 6.27 ± 0.07%, which were significantly higher in ethylene ripened mangoes (8.26 ± 0.02%), and carbide ripened mangoes (9.90 ± 0.03%). Highest amount of protein and fiber was noticed in mangoes ripened with 500 µL/L ethylene. Lower fat contents were observed in naturally ripened mangoes as compared to artificially ripened, in which contents of fat were found slightly increased with increased concentrations of ripening agents.

**Table 2:** Proximate composition of mangoes, naturally ripened, ripened with calcium carbide and artificial ethylene.

Proximate composition of mangoes ripened through different techniques							
(a)	Concentration (g/kg)	Moisture (%)	Ash (%)	Fat (%)	Fibre (%)	Protein (%)	Carbohydrates (%)
Mature mangoes ripened with calcium carbide	1	78.36 ± 0.08c	4.94 ± 0.02c	2.65 ± 0.01c	3.05 ± 0.02bc	3.23 ± 0.04b	7.83 ± 0.05e
	5	77.53 ± 0.06d	5.27 ± 0.01b	2.77 ± 0.03b	2.95 ± 0.06c	3.12 ± 0.04c	8.36 ± 0.06b
	10	75.85 ± 0.06e	5.46 ± 0.01a	2.99 ± 0.02a	2.51 ± 0.04d	3.03 ± 0.03c	9.90 ± 0.03a

(b)	Concentration (µL/kg)	Moisture	Ash	Fat	Fibre	Protein	Carbohydrates
Mature mangoes ripened with artificial ethylene	500	79.45 ± 0.08b	4.19 ± 0.04e	2.35 ± 0.01d	3.49 ± 0.03a	3.49 ± 0.02a	6.92 ± 0.07f.
	1000	78.23 ± 0.05c	4.69 ± 0.01d	2.60 ± 0.03c	3.15 ± 0.04b	3.30 ± 0.04b	8.04 ± 0.06d
	1500	77.63 ± 0.06d	5.15 ± 0.02b	2.77 ± 0.02b	3.05 ± 0.01bc	3.12 ± 0.05c	8.26 ± 0.02c

(c)	Moisture	Ash	Fat	Fibre	Protein	Carbohydrates	
Mature mangoes ripened naturally	80.21 ± 0.09a	4.74 ± 0.03d	2.10 ± 0.04e	3.57 ± 0.04a	3.05 ± 0.05c	6.27 ± 0.07 g	

Each value is the Mean ± SD. Means with similar alphabetical letters in a column indicates non-significant results, whereas means with different alphabetical letters in a column indicates significant results (p < 0.05).

### Organoleptic parameters of mangoes, naturally ripened, ripened with calcium carbide and artificial ethylene

From the results summarized in Table 3, significantly high (*p* < *0.05*) scores for taste (8.12 ± 0.03), aroma (8.62 ± 0.07), firmness (8.43 ± 0.06) and overall acceptability (8.50 ± 0.05) can be seen for naturally ripened mangoes, while the significantly low (*p* < *0.05*) scores for same parameters were recorded for mangoes ripened with 10 g/kg application of calcium carbide, which indicates the deleterious effects of calcium carbide on ripening of mangoes. But for the color of mangoes highest scores (7.90 ± 0.06) were given to calcium carbide ripened mangoes at higher concentrations, which was due to the reason that calcium carbide imparts brighter yellow to orange color to the mangoes uniformly, in short period of time. Higher concentrations of ethylene also provided good organoleptic scores to the mangoes as synthetic ethylene is also capable to impart uniform color and ripening of mangoes, whereas other parameters, like taste and aroma have been found affected negatively. At the end, comparing overall acceptability of mangoes, higher acceptability scores were found for ethylene ripened mangoes as compared to carbide ripened mangoes.

**Table 3:** Organoleptic parameters of mangoes, naturally ripened, ripened with calcium carbide and artificial ethylene.

Organoleptic parameters of mangoes ripened through different techniques							
(a)	Concentration (g/kg)	Color	Aroma	Taste	Off odour	Firmness	Overall acceptability
Mature mangoes ripened with calcium carbide	1	6.65 ± 0.03c	8.06 ± 0.03b	6.09 ± 0.02e	5.12 ± 0.02d	7.45 ± 0.03c	6.82 ± 0.03d
	5	7.20 ± 0.04b	6.42 ± 0.06d	5.12 ± 0.03f	4.74 ± 0.02e	6.86 ± 0.02d	6.23 ± 0.02e
	10	7.90 ± 0.06a	5.06 ± 0.06e	4.35 ± 0.06g	4.49 ± 0.03f	5.37 ± 0.04e	5.21 ± 0.05f

(b)	Concentration (µL/kg)	Color	Aroma	Taste	Off odour	Firmness	Overall acceptability
Mature mangoes ripened with artificial ethylene	500	5.94 ± 0.06d	7.35 ± 0.05c	6.35 ± 0.03d	6.49 ± 0.04c	7.41 ± 0.05c	7.60 ± 0.02c
	1000	6.61 ± 0.06c	7.80 ± 0.02b	6.85 ± 0.04c	6.60 ± 0.04bc	7.79 ± 0.03b	8.09 ± 0.03b
	1500	7.15 ± 0.02c	8.45 ± 0.05a	7.83 ± 0.07b	6.73 ± 0.03b	8.26 ± 0.02a	8.53 ± 0.06a

(c)	Color	Aroma	Taste	Off odour	Firmness	Overall acceptability	
Mature mangoes ripened naturally	7.29 ± 0.05b	8.62 ± 0.07a	8.12 ± 0.03a	8.35 ± 0.05a	8.43 ± 0.06a	8.50 ± 0.05a	

Each value is the Mean ± SD, n = 80. Means with similar alphabetical letters in a column indicates non-significant results, whereas means with different alphabetical letters in a column indicates significant results (p < 0.05).

### Total soluble solids, titratable acidity, reducing sugars, ascorbic acid, citric acid and malic acid contents of mangoes, naturally ripened, ripened with calcium carbide and artificial ethylene

From the data presented in Table 4, significant (*p* < *0.05*) increase in total soluble solids of the mangoes was noticed, with the increased concentrations of ethylene and calcium carbide, while highest total soluble solids (15.78 ± 0.04 Brix) were recorded in mangoes, applied with 10 g/kg calcium carbide. Brix of naturally ripened mangoes was recorded 14.10 ± 0.01, which was quite lower than of artificially ripened mangoes. Titratable acidity of naturally ripened mangoes (0.31 ± 0.01%) was higher as compared to mangoes ripened with higher levels of ethylene and calcium carbide, as value of titratable acidity for both treatments was found 0.29 ± 0.02%. Significant (*p* < *0.05*) reduction in titratable acidity of mangoes was observed with increasing the concentrations of artificial ripening agents. Results of reducing sugars indicated significant (*p* < *0.05*) increase in reducing sugars with increased levels of application of ethylene and carbide as external ripening chemicals. Significantly high (*p* < *0.05*) reducing sugars (7.35 ± 0.02%), were recorded in mangoes ripened with higher levels of ethylene, followed by in mangoes ripened with higher levels of carbide (7.19 ± 0.04%), while in naturally ripened mangoes percentage of reducing sugars was observed 6.60 ± 0.03%.

**Table 4:** Total soluble solids, titratable acidity, reducing sugars, ascorbic acid, citric acid and malic acid contents of mangoes, naturally ripened, ripened with calcium carbide and artificial ethylene.

Chemical constituents of mangoes ripened through different techniques							
(a)	Concentration (g/kg)	Total soluble solids (Brix)	Titratable acidity (%)	Reducing sugar (%)	Ascorbic acid (mg/g)	Citric acid (mg/g)	Malic acid (mg/g)
Mature mangoes ripened with calcium carbide	1	14.20 ± 0.01e	0.36 ± 0.01a	5.20 ± 0.03f	49.49 ± 0.03b	3.29 ± 0.02cd	0.97 ± 0.01c
	5	15.05 ± 0.02b	0.32 ± 0.01ab	6.97 ± 0.04b	43.27 ± 0.07e	2.75 ± 0.03e	0.78 ± 0.02d
	10	15.78 ± 0.04a	0.29 ± 0.02b	7.35 ± 0.02a	35.94 ± 0.04g	02.12 ± 0.02f	0.63 ± 0.01e

(b)	Concentration (µL/kg)	Total soluble solids (Brix)	Titratable acidity (%)	Reducing sugar (%)	Ascorbic acid (mg/g)	Citric acid (mg/g)	Malic acid (mg/g)
Mature mangoes ripened with artificial ethylene	500	13.95 ± 0.01g	0.33 ± 0.04ab	5.45 ± 0.02e	45.26 ± 0.04c	3.54 ± 0.02b	1.02 ± 0.02b
	1000	14.30 ± 0.04d	0.31 ± 0.01b	6.38 ± 0.01d	43.87 ± 0.06d	3.44 ± 0.05bc	1.17 ± 0.03b
	1500	14.86 ± 0.02c	0.29 ± 0.02b	7.19 ± 0.04a	40.28 ± 0.03f	3.22 ± 0.03d	1.06 ± 0.02c

(c)	Total soluble solids (Brix)	Titratable acidity (%)	Reducing sugar (%)	Ascorbic acid (mg/g)	Citric acid (mg/g)	Malic acid (mg/g)	
Mature mangoes ripened naturally	14.10 ± 0.01f.	0.31 ± 0.01b	6.60 ± 0.03c	52.29 ± 0.05a	3.76 ± 0.06a	1.37 ± 0.04a	

Each value is the Mean ± SD. Means with similar alphabetical letters in a column indicates non-significant results, whereas means with different alphabetical letters in a column indicates significant results (p < 0.05).

Evaluation of ascorbic acid, citric acid and malic acid, in mangoes, ripened through different ways, is presented in Table 4. Data shows the significantly high (*p* < *0.05*) retention of these acids, with values 52.29 ± 0.05, 3.76 ± 0.06 and 1.37 ± 0.04 mg/g, respectively, in naturally ripened mangoes. Ripening with higher concentrations of ethylene and calcium carbide, resulted significant (*p* < *0.05*) reduction in the amounts of these organic acids. Although decrease of these acids is natural process during ripening of mangoes, but reduction due to application of calcium carbide was more prominent, which might be due to increased respiration and metabolism in tissues.

### Minerals composition of mangoes naturally ripened, ripened with calcium carbide and artificial ethylene

Data of minerals analysis presented in Table 5 revealed significantly higher (*p* < *0.05*) concentrations of minerals in naturally ripened mangoes, as compared to the calcium carbide ripened mangoes. Amounts of P, K, Mg, Fe, Zn, Ca and Cu, in naturally ripened mangoes were 18.85 ± 0.04, 135.61 ± 0.06, 13.28 ± 0.05, 0.45 ± 0.04, 0.24 ± 0.02, 8.89 ± 0.05 and 0.17 ± 0.02 mg/100 g mangoes, respectively, while in mangoes, with applied concentration of calcium carbide at 10 g/kg, amounts of these minerals were recorded 15.56 ± 0.05, 121.51 ± 0.06, 11.32 ± 0.03, 0.29 ± 0.04, 0.12 ± 0.01, 7.99 ± 0.03 and 0.11 ± 0.01 mg/100 g, respectively. Significant (*p* < *0.05*) reduction in the mineral contents was noticed with the increased concentration of calcium carbide, whereas significant (*p* < *0.05*) increment in the minerals, was witnessed with increasing the concentration of ethylene, as an external ripening agent.

**Table 5:** Minerals composition of mangoes naturally ripened, ripened with calcium carbide and artificial ethylene.

Mineral composition of mangoes ripened through different techniques								
(a)	Concentration (g/kg)	Minerals (mg/100 g)						
		P	K	Mg	Fe	Zn	Ca	Cu
Mature mangoes ripened with calcium carbide	1	16.39 ± 0.02e	126.67 ± 0.05d	12.41 ± 0.02d	0.36 ± 0.03d	0.15 ± 0.01cd	8.47 ± 0.02d	0.14 ± 0.01bc
	5	15.90 ± 0.03f	124.27 ± 0.05e	12.07 ± 0.05e	0.33 ± 0.02e	0.13 ± 0.02d	8.12 ± 0.06e	0.12 ± 0.01c
	10	15.56 ± 0.05g	121.51 ± 0.06f	11.32 ± 0.03f	0.29 ± 0.04e	0.12 ± 0.01d	7.99 ± 0.03e	0.11 ± 0.01c

(b)	Concentration (µL/kg)	P	K	Mg	Fe	Zn	Ca	Cu
Mature mangoes ripened with artificial ethylene	500	17.56 ± 0.03d	131.21 ± 0.06c	12.95 ± 0.05c	0.39 ± 0.03c	0.15 ± 0.03 cd	8.81 ± 0.03c	0.15 ± 0.02abc
	1000	18.61 ± 0.06c	133.48 ± 0.07b	13.45 ± 0.04b	0.45 ± 0.05b	0.17 ± 0.01b	9.57 ± 0.02b	0.16 ± 0.02ab
	1500	19.27 ± 0.07a	136.01 ± 0.08a	14.49 ± 0.06a	0.52 ± 0.02a	0.20 ± 0.01b	10.24 ± 0.05a	0.18 ± 0.01a

(c)	P	K	Mg	Fe	Zn	Ca	Cu	
Mature mangoes ripened naturally	18.85 ± 0.04b	135.61 ± 0.06a	13.28 ± 0.05a	0.45 ± 0.04b	0.24 ± 0.02a	8.89 ± 0.05c	0.17 ± 0.02a	

Each value is the Mean ± SD. Means with similar alphabetical letters in a column indicates non-significant results, whereas means with different alphabetical letters in a column indicates significant results (p < 0.05).

## Discussion

Similar observations were provided by Sogo-Temi et al.^25^, during their studies of the effects of natural ripening and chemical agents-based ripening techniques, on sensory, physical and chemical characteristics of the fruits, validating the deleterious effects of calcium carbide on physicochemical parameters of the mango. Artificially used ripening and preserving agents have been frequently reported in different food poisoning cases in different developing and underdeveloped countries, due to their adverse health effects. These ripening agents not only affect the physical structures of the fruits, but also poses negative effects on the nutritional status of the fruits and fruit-based food products^26^. Therefore, natural ripening may be preferred over artificial, for mangoes. Adeyemi et al.^27^ studied the effect of application of calcium carbide at different concentrations to ripe three different fruits (banana, mango and papaya) and calculated the shelf life, and ripening times of fruits under study. The observations supported the findings of present research work, as without use of calcium carbide, both shelf life and ripening time were greater, whereas as the concentration of ripening agent was increased, both shelf life and ripening time were decreased. Pandarinathan and Sivakumar^28^ also noticed decrease in the days for ripening of mangoes, applied with calcium carbide, providing results in line with current ones. In another study, reporting similar results, Palpandian et al.^29^ concluded that use of calcium carbide provides early ripening of treated mangoes, but shelf life got decreased. Mamiro et al.^30^ made experiments on mangoes tested for room temperature ripening, ripening by smoke and ripening by ethylene, and unveiled the facts that ripening of mangoes was geared by natural ethylene.

Natural ethylene produced by fruits itself, artificial ethylene applied externally, and use of synthetic calcium carbide, all affects the mangoes’ gas composition, as mangoes are undergone respiratory, catalytic and climacteric changes, which results in difference of ripening time, and shelf-life of mangoes, under study. It has also been suggested that, the faster ripening of detached fruit may be explained by changes in the fruit’s water balance, and skin’s resistance to gas diffusion, caused by fruit detachment^9^. Ripening process was correlated with temperature of post-harvest storage, by Diop et al.^31^, and investigations revealed that temperature is directly related with ripening time, and shelf life of the mangoes, and use of synthetic chemicals as ripening agent increase the temperature of the containers, holding mature mangoes, forcing their speedy ripening, just as happened in current trials. Supportive results were also found in the findings of Abbas et al.^32^, when they studied the effect of use of calcium carbide as ripening agent at different concentrations for mangoes, reporting that calcium carbide might provide speedy ripening, but with compromised shelf life.

Adeyemi et al.^27^ analyzed the proximate composition of three fruits ripened at different concentrations of calcium carbide, and similar results were observed for moisture, ash, fat, fiber, protein and carbohydrates contents. They reported that increase in lipid contents might be due to the increased respiration mechanism, which ultimately would have been resulted in biosynthesis of lipids. Result of present study were also in close resemblance with the observations of the Aberoumand^33^. In another study, Imran et al.^34^ collected commercially available different mango varieties artificially ripened, from the local markets of Pakistan and determined their chemical composition. The results were comparable with the findings of present research work, for the treatments ripened with commercially available chemical reagents. The amounts of moisture, protein, fat, fiber, ash and NFE were found in the range of 68.33–71.38, 1.94–2.36, 2.11–2.31, 4.53–5.01, 1.84–2.59 and 87.60–89.58 g/100 g, respectively. Langeh et al.^35^ studied the effect of different pretreatments on physicochemical composition of mangoes, and significant variations in the results were found for moisture, ash and fiber contents. Jean-Claude et al.^36^ made a comparison physicochemical composition of mature green unripe mangoes with fully ripened mangoes. The percentage of moisture, ash, protein and fiber in fully ripened mangoes was found 77.09, 0.32, 0.70 and 1.53 respectively, in naturally ripened mangoes, and these values were comparable with the current findings.

Mamiro et al.^30^ conducted research experiments on mangoes tested for room temperature ripening, ripening by smoke and application of plant-based ethylene, significant difference in moisture contents was found as moisture content in ethylene-based ripened mangoes was lesser as compared to untreated mangoes. Similarly, amount of crude fiber was lesser in ethylene-based mangoes as compared to naturally ripened mangoes at room temperature. Non-significant results were observed for protein and fat contents for all types of mangoes, while carbohydrates were decreased in ethylene-based ripened mangoes as compared to room temperature-based mangoes. Pandarinathan and Sivakumar^28^ noticed a decrease in the protein contents of mangoes ripened with calcium carbide. They concluded that loss of carbon and water may be occurred due to the reason of respiration and transpiration processes, respectively during storage and ripening period. Decrease in insoluble pectin and increase in soluble pectin in the course of ripening might be the reason of decrease in crude fiber contents of artificially ripened mangoes. Slight increase in crude fat contents might be linked with color and flavor development of mangoes during ripening as biosynthesis of triglycerides and fatty acids may be encouraged by presence of acetyl coenzyme A, during extended storage. On the other hand, increase in protein contents in artificially ripened mangoes have been found associated with increased amounts of enzymes during ripening^30^.

Although calcium carbide hastens the ripening process of the mangoes and provide attractive color but consumption of these artificially ripened mangoes poses serious health hazards, especially changes in hematological and biochemical profiles of the animals and are capable of inducing liver, kidney and immune related diseases^16^. Use of calcium carbide has always adverse effects on physicochemical and nutritional profiles of the mangoes, as such fruits have been found to contain arsenic residues in peel and pulp fractions, which should be free from such heavy metals^37^. In the findings of Abbas et al.^32^, deterioration in organoleptic parameters of the artificially ripened mangoes was observed, as compared to the naturally ripened mangoes, as use of calcium carbide as ripening agent caused loss of firmness of the skin, dark and yellow patches, less attractiveness and shivering on skin. Changes in skin and pulp color of the fruits were noticed during the storage period of artificially ripened mangoes, under controlled conditions. It was observed that loss of firmness in calcium carbide ripened fruits was faster than in the ethylene ripened fruits^38^. Rapid off-tree ripening of mangoes are correlated with the loss of firmness and excessive softening during storage period, which ultimately reduces the required level of marketing of this precious fruit^10^. Natural ripening process positively affects the physiological and biochemical alteration in the mangoes, which includes, color changes, dehydration, cellular respiration, transformation of organic acids and polysaccharides, resultantly contributing towards good scores for organoleptic features of the mangoes^39^.

Findings of Zhang et al.^8^ also revealed that firmness of the artificially ripened fruits got decreased with increasing the concentration of the ripening agents and with the passage of time, during storage period. Montalvo et al.^17^ applied exogenous ethylene to the mangoes at 100, 500 and 1000 µL/kg mangoes, and compared these with the control mangoes naturally ripened at room temperature, and observed uniform color development of ethylene provided mangoes but slight loss of firmness, while overall acceptability of mangoes was good. In the findings of Tovar et al.^40^, application of exogenous ethylene at higher concentrations, as an artificial ripening agent was proved useful in 100% development of uniform color of peel and pulp. Mangoes ripened by external ethylene were compared with naturally ripened mangoes, at room temperature, in which uniformity of color development was not obvious, just as was seen in current experiments that natural ripening at room temperature failed to impart uniform color to the mangoes. However, the taste, flavor, aroma and overall acceptability of naturally ripened mangoes were comparatively high, as compared to those ripened artificially.

Pandarinathan and Sivakumar^28^ studied the effect of application of calcium carbide as ripening agent, at different concentrations on different varieties of mangoes, and noticed significant decrease in ascorbic acid, reducing sugars and total soluble solids as a result of increasing the concentration of calcium carbide, supporting the current findings. Palpandian et al.^29^ performed chemical analyses of controlled and calcium carbide ripened mangoes, and results were also in line with our findings. In another study, Silva et al.^38^ compared the quality parameters of mangoes ripened with artificial ethylene and calcium carbide and observed increased mass loss of the fruits due to increased respiratory changes, which was observed due to the higher metabolism in tissues and cells. Total soluble solids were found increased in mangoes ripened with ethylene as compared to calcium carbide. Titratable acidity was reduced in mangoes both ripened with calcium carbide and ethylene. Ascorbic acid contents were found decreased in mangoes ripened by both methods, which was probably due to the increased electrolyte leakages. Naturally occurring antioxidants in fruits, have been found involved in promoting human health, but the use of artificial ripening techniques adversely affects these naturally present components, as use of calcium carbide significantly reduced the vitamin C contents of the mangoes^27^. Iheagwam et al.^41^ calculated phytochemical screening and vitamin contents in unripe, naturally ripened and artificially ripened mangoes, vitamin C was found 51.06 mg/100 g in unripe mangoes, which was deceased to 41.54 mg/100 g in calcium carbide ripened mangoes, whereas in naturally ripened mangoes contents of vitamin C were found 30.90 mg/100 g.

In another similar study, Diop et al.^31^ compared the total soluble solids, pH and titratable acidity of mature green and naturally ripened mangoes, and observed a significant increase in pH and total soluble solids of naturally ripened mangoes, as compared to mature green mangoes, whereas titratable acidity was decreased during ripening. Further, conversion of starch into soluble sugars, reduction in citric acid contents, and lower titratable acidity values were found in naturally ripened mangoes, just as happened in current trials. Langeh et al.^35^ reported that reducing sugars increase with the increase in storage period. This could be due to the hydrolysis of sugars, which might be due to the degradation of disaccharides to monosaccharides. They also reported that decrease in ascorbic acid during storage, might be due to the increase in the moisture content, as well as atmospheric temperature and oxygen, and presence of trace metals. Montalvo et al.^17^ applied exogenous ethylene to the mangoes at same concentrations, used in our study, and supportive results were found, indicating significant increase in TSS and decrease in titratable acidity of mangoes, ripened at higher concentration of exogenous ethylene. In the findings of Tovar et al.^40^, concomitant increase in the respiration rate and total soluble solids, with proceeded loss of acidity and firmness, was recorded, as a result of exogenous ethylene at a rate of 1500 µL/kg.

Ripening causes three-to-four-fold decrease in titratable acidity, from mature green to fully ripened mangoes, and reduction of acidity of fruits during ripening is a natural phenomenon, which is affected by several factors, but the most relevant is increase in sweetness of fruits. The decrease in ascorbic acid and other organic acid contents is an attribute of oxidative destruction of the acids during ripening. Whereas, increase in total soluble solids might be due to the degradation of large pectin molecules, and hydrolytic conversion of starch yielding free sugars^30^. In the soft tissues of mangoes, the lower contents of reducing sugars can be engaged to the decreased activities of the amylase and invertase enzymes. The stats show that during ripening of the mangoes, amylolytic activities play an important role on concentrations of sugars^20^.

Organic acids in the fruits have been characterized due to their weaker acidic capacities, aerobic metabolism, and as a part of flavoring compounds, which impart aroma, taste, flavor and acidity to the fruits. Major contributors towards acidity of mangoes, after ascorbic acid, are known citric and malic acids, but their concentrations have been found different in mangoes depending upon different pre-harvest and post-harvest treatments, along with the cultivars^5^. Mango pulp contains various organic acids including citric acid, malic acid, oxalic acid, succinic acid, ascorbic acid, and tartaric acid. Organic acids are generally weak acids with low molecular weight^42^. Decrease in acidity of mangoes during ripening may be due to the loss of citric acid and malic acid, during ripening process^43^. During the HPLC analysis of mango pulp samples, by Tovar et al.^22^, concentrations of the weak organic acids were found varying, as a result of different post-harvest treatments to the mango slices. Thus, optimum retention of the naturally occurring components of fruits, could only be achieved through natural ripening.

Mango pulp is a good source of minerals, that are essential for a variety of biochemical reactions. The consumption of mango provides a considerable amount of many micro- and macrominerals, such as calcium, sodium, copper, iron, phosphorus, manganese, magnesium and zinc^11^. However, mangoes ripened with calcium carbide got entry of purities, arsine and phosphine, while these metal residues have been found accumulated in peel and pulp of such mangoes^15^. Potential health hazards associated with use of calcium carbide have been listed as dizziness, sleepiness, mood disturbance, headache, mental confusion, seizures, diarrhea, cancer and neurological disorders^44^. Results of Hernandez-Sanchez et al.^24^ were very helpful in comparing the values of mineral contents of present study, with the average values of minerals, found in different conventional and organically grown mangoes. Mean values of Cu, Zn, Fe, Ca, Mg, Na and K in conventionally grown ripened mangoes were found 0.07, 0.20, 1.97, 47.30, 18.08, 11.44 and 146.60 mg/100 g, respectively. They observed a significant difference in mineral contents of organically grown mangoes as compared to conventional mangoes, which revealed that cultivations conditions also affect the mineral composition of the mangoes.

Abubakar^14^ studied the effect of application of calcium carbide at 0, 1, 5 and 10 g/kg mangoes, on mineral composition, and observed a slight increase in trace elements of mangoes, ripened at lower concentration of calcium carbide, but a significant decrease in minerals quantity was observed in fruits ripened at 10 g/kg application of calcium carbide, just as was observed in current results. In another similar study, Khan et al.^12^ performed elemental analysis of mangoes ripened through different post-harvest techniques, including use of ethylene, calcium carbide and natural ripening. Contents of Ca, Mg and K were found significantly different in all mangoes, ripened with different adopted techniques, just in accordance with the current findings. According to Mamiro et al.^30^, different ripening environments, induce different metabolic reactions, causing the variation in chemical makeup of the mangoes. Jean-Claude et al.^36^ conducted the evaluation of mineral composition of unripe and naturally ripened mangoes, and values of Ca, Mg, K, P, Fe, Zn and Cu were found 21.24, 28.70, 249.16, 22.15, 1.48, 0.03 and 0.11 mg/100 g of mango pulp, respectively in fully ripened mangoes. Their results validated the current ones, as a significant increase in mineral concentrations was observed during natural ripening process of mangoes as amounts of these minerals were found lower in mature unripe mangoes. In another similar study, with supportive results, Imran et al.^34^ determined minerals composition of different commercially ripened mangoes varieties found in market. Amounts of K, Mg, Ca and Fe were found in the range of 16.21 to 18.78, 50.25 to 56.83, 75.08 to 87.46 and 5.35 to 8.83 mg/100 g, respectively, where amounts of Cu, Mn and Cr were found 0.056 to 0.076, 0.036 to 0.046 and 0.26 to 0.27 mg/100 g, respectively. Andrew et al.^16^ conducted experiments on animals, fed with mangoes artificially ripened by use of calcium carbide, and observed a significant decrease in potassium, calcium and bicarbonate levels. The imbalance of these electrolytes was possibly due to the toxic effects of calcium carbide. The electrolytes balance in the body is used as an indication of normal body functions, like hormonal balance, kidney functioning, body hydration and lungs working. Therefore, natural ripening could be promoted for mangoes, as these mangoes could provide high mineral contents, with minimum toxic elements.

## Conclusion

Although the use of calcium carbide can provide rapid ripening of fruits to meet the increasing demand of populations, but the use of such types of artificial chemical-based ripening and preservation agents always results in adverse effects on the physical, chemical, nutritional and sensorial characteristics of the fruits. In the present study a comparison of three different ripening techniques demonstrated that natural ripening is the best ripening process. With natural ripening, there was maximum shelf-life retention as well as optimum product quality of the mangoes with maximum retention of nutrients and minimum toxicity. Ripening with commercial ethylene although provided acceptable quality mangoes but due to its high cost, this technique is challenging for local growers and farmers. On the other hand, calcium carbide provided rapid ripening of mangoes with uniform and attractive color development, but the physicochemical nature of the mangoes was adversely affected. Consequently, there is need is to develop and implement accessible, affordable innovative technologies that can retain the maximum natural bioactives of mangoes with minimum quality deterioration without the use of synthetic chemicals.

## Recommendations

Mango, also known as king of the fruits, is the main produce of Asian subcontinent and especially Pakistan, as it is best source of carbohydrates, proteins, fatty acids, vitamins, minerals, polyphenols, phenolics, flavonoids and carotenoids. Due to improper post-harvest handling, recently the use of synthetic ripening agents and distancing from the natural products has raised consumer concerns regarding health impairments related to the consumption of these fruits. Natural ripening of mangoes should be encouraged in order to support the enhanced nutrition that this remarkable fruit offers. However, more pharmacokinetics, pharmacodynamics, and clinical trials are warranted in order to assess the toxicity and minimal safety parameters of these synthetic ripening agents. Nevertheless, overall mango is considered safe and is beneficial for cellular functioning if proper post-harvest and ripening techniques are adopted. Regular consumption of freshly ripened mango fruit and/or use of its byproducts and food products could promote overall health.

## Data Availability

Data relevant to this study can be provided by submitting author, Ashiq Hussain (ashiqft@gmail.com), upon request.
